# Working with AI: The Effect of Job Stress on Hotel Employees’ Work Engagement

**DOI:** 10.3390/bs14111076

**Published:** 2024-11-11

**Authors:** Yong Hou, Liwei Fan

**Affiliations:** 1 School of Economics and Management, Weifang University of Science and Technology, Weifang 262700, China; houyong@wfust.edu.cn; 2 School of Management, Xiamen University, 422, Siming South Road, Xiamen 361005, China

**Keywords:** AI-induced stress, psychological capital, work engagement, perceived organizational support

## Abstract

Based on the Conservation of Resources (COR) theory and social support theory, this study focuses on the effects of AI-induced stress on hotel employees’ work engagement and examines the mediating role of psychological capital and the moderating role of perceived organizational support. A sample of five-star hotels in China was selected for the study, data were analyzed, and hypotheses were tested using SPSS 27.0 and Mplus 7.4 software. The results of the study revealed that AI-induced stress had a significant negative effect on work engagement and psychological capital mediated the relationship between AI-induced stress and work engagement. Perceived organizational support moderated the relationship between work stress and psychological capital. Specifically, the higher the perceived organizational support, the lower the negative effect of work stress on psychological capital; conversely, the lower the perceived organizational support, the higher the negative effect of work stress on psychological capital. The greater the negative impact of work stress on psychological capital, the higher the perceived organizational support, and the smaller the negative impact of work stress on psychological capital. The findings of the study not only enrich the research related to AI in the hotel industry but also have certain reference significance for managers in the hotel industry who introduce AI in managing their employees.

## 1. Introduction

With the widespread popularization and application of cutting-edge generative AI technologies, such as DALL-E and ChatGPT, AI-induced stress has become more and more prominent and a common challenge faced by many practitioners [1,2]. The rapid advancement of technology and increasing competition have made the hotel industry’s work environment more complex and challenging. Automation and artificial intelligence (AI) have transformed customer service, enabling hotels to use chatbots and virtual assistants for inquiries and reservations, which enhances efficiency and allows employees to focus on personalized service. Data analytics helps optimize marketing strategies by analyzing customer preferences, while smart room technology, facilitated by the Internet of Things (IoT), enhances guest comfort but requires staff to operate these systems effectively. The rise of online booking platforms has revolutionized reservation management, necessitating that employees adapt to modern property management systems. Additionally, social media and online reviews demand prompt responses from hotels to maintain their brand image, adding pressure on staff. Finally, the adoption of mobile payment solutions and contactless services enhances convenience but requires employees to become proficient in new technologies. As a result, while these advancements aim to improve service quality and customer satisfaction, they also present new challenges that employees must navigate in an ever-evolving work environment [3,4]. AI-induced stress has many effects on physical and mental health and the performance of hotel employees, and it also has a series of impacts on organizational managers [5]. According to the “China Workplace Stress Report 2021”, the stress index of workplace people in the first half of 2021 is as high as 7.26, which is a record high in the past four years. Among them, the 25–30-year-old workplace group has the strongest sense of stress. The higher the income of employees with an annual income of more than CNY 400,000, the stronger the sense of stress. Therefore, AI-induced stress is a common problem in modern society and affects the normal operation of organizations to a certain extent.

In the hospitality industry, AI-induced stress is defined as the psychological stress and emotional burden on employees or managers due to the application of AI technology [6]. It has been found that AI-induced stress increases the likelihood of negative work behaviors among hotel employees [6]. On the one hand, there is a significant positive correlation between work stress and negative work behaviors among hotel employees [7,8]. At the same time, AI-induced stress may lead to increased emotional instability and negativity among hotel employees, which, in turn, may motivate them to adopt behaviors that are harmful to the organization [9]. Work engagement refers to the positive, energetic, and focused mental state that employees display at work [10]. Work engagement of hotel employees is not only an important guarantee for the development of enterprises, but it also directly affects the core competitiveness and sustainable development of enterprises [10]. Therefore, it is of great significance to investigate the mechanism of AI-induced stress on work engagement.

People are always actively working to maintain, protect, and build what they consider to be valuable resources. This proactive behavior stems from the inherent human instinct to safeguard against the potential or actual loss of these resources, which can pose a significant threat to their well-being and stability [11]. Among these resources, psychological capital stands out as a vital asset, encompassing an individual’s psychological state characterized by self-efficacy, optimism, resilience, and hope. Importantly, psychological capital is not only an individual resource but also an organizational resource, serving as a foundational pillar that contributes to the overall development and performance of an organization. When employees possess high levels of psychological capital, they are more inclined to engage in innovative thinking, effective problem-solving, and cooperative teamwork, all of which are essential for organizational success. Furthermore, organizations that foster a culture of psychological capital not only improve employee satisfaction and retention but also enhance their adaptability in dynamic market environments. Consequently, understanding and nurturing psychological capital becomes crucial for organizations seeking to thrive in an increasingly competitive landscape, as it directly influences their ability to achieve strategic goals and sustain long-term growth. Research has shown that when individuals face work stress, they may feel negative emotions such as anxiety, fatigue, and helplessness, which may reduce their work engagement and performance [12,13]. However, if individuals have certain psychological capital, they can better cope with work stress and, thus, improve their work engagement and performance. Because human potential is huge, and the appreciation potential of psychological capital far exceeds that of financial, market, and technological capital, superior psychological capital will become a decisive competitive advantage [14,15]. Therefore, it is important for this study to explore the mechanisms through which AI-induced stress affects hotel employees’ work engagement and to clarify whether psychological capital mediates the relationship between AI-induced stress and work engagement.

Social support theory suggests that adequate social support can enhance individuals’ resilience and adaptability, reduce the occurrence of mental health problems, and improve the quality of life [16]. Specifically, in the workplace context, perceived organizational support plays a crucial role in providing emotional support, fostering a sense of belonging, and creating a positive work environment that can significantly mitigate stressors. By reinforcing an individual’s sense of identity with the organization, perceived organizational support can be particularly effective in alleviating the negative effects of AI-induced stress on psychological capital, which encompasses an individual’s mental resources, including self-efficacy, hope, optimism, and resilience [17]. Moreover, as organizations increasingly integrate artificial intelligence into their operations, the stress related to navigating this technological shift may pose unique challenges to employees’ psychological well-being. Therefore, the present study explored whether AI-induced stress negatively affects psychological capital and examined the potential moderating effect of perceived organizational support on this relationship. Understanding these dynamics is essential for developing interventions aimed at enhancing employee well-being and fostering a resilient workforce capable of adapting to the rapid advancements in technology.

In summary, this study intends to investigate the mechanism of AI-induced stress on employees’ work engagement in five-star hotels and to test the mediating role of psychological capital and the moderating role of perceived organizational support. This study can ultimately provide relevant practical insights and theoretical basis for companies to choose how to mitigate the negative effects of AI-induced stress on hotel employees’ work engagement so as to improve hotel employees’ performance and the organization’s operational capability.

## 2. Research Hypotheses

### 2.1. AI-Induced Stress and Work Engagement

Based on the COR theory, individuals deplete psychological and emotional resources when facing stress, and work engagement requires sufficient resource support [11]. AI-induced stress may lead to resource depletion in individuals, including psychological and emotional resources. When individuals face high levels of stress, resource depletion may exceed their regenerative capacity, leading to resource depletion [9,18]. At the same time, work engagement requires individuals to have sufficient resources, including psychological and emotional resources. Work engagement requires individuals to maintain focus, positive emotions, and a high level of commitment, all of which need to be supported by sufficient resources. When individuals face resource depletion, they may not be able to engage effectively [19]. As a result of resource deprivation, individuals may feel tired, negative, or unable to concentrate, leading to a decrease in work engagement. At the same time, work engagement may also have an impact on resource consumption. High levels of work engagement may increase an individual’s resource depletion, especially if the high level of engagement is sustained over a long period of time, which may lead to rapid resource depletion [20].

Thus, there is an interactive relationship between AI-induced stress and work engagement. Stress may lead to the depletion of resources, which may decrease work engagement [21]. Work engagement itself may increase resource depletion [22]. The result of this interaction may be a negative cycle. That is, stress leads to resource depletion, which, in turn, decreases work engagement, and decreased work engagement further increases resource depletion, exacerbating feelings of stress [23]. At the same time, researchers believe that individuals’ behavior is not determined by either internal or external environmental factors alone but is altered by a combination of both. AI-induced stress is regarded as an external stimulus from the environment, while work engagement is a positive response to the work environment [24]. When individuals perceive higher levels of AI-induced stress, it may lead to a decrease in work engagement among hotel employees [25]. Therefore, there is a negative relationship between AI-induced stress and work engagement, i.e., when AI-induced stress increases, individuals may be more negatively engaged. Based on the above theoretical analysis, this study proposed the following hypothesis.

**Hypothesis** **1.** *AI-induced stress has a significant negative effect on work engagement*.

### 2.2. The Mediating Role of Psychological Capital Between AI-Induced Stress and Work Engagement

Psychological capital refers to a person’s positive psychological state, which mainly includes four aspects such as self-confidence, hope, optimism, and resilience [26]. Psychological capital affects a person’s state, which, in turn, affects his or her behavior. Environment, individual, and behavior are interrelated, and environment and personality traits affect behavior, and behavior, in turn, causes changes in the environment and the individual [27]. When individuals feel work pressure, they will make negative subjective judgments and produce negative effects, such as emotional exhaustion [28]. According to the COR theory, individuals strive to acquire and protect their resources, including psychological assets such as self-efficacy, resilience, optimism, and hope. AI-induced stress can deplete these vital psychological resources by overwhelming employees with excessive demands for adaptation, leading to feelings of inadequacy and decreased confidence in their abilities. As stress levels rise, employees may experience anxiety and frustration, which can undermine their optimism and resilience, creating a cycle of resource depletion. Consequently, the negative impact of AI-induced stress on psychological capital becomes evident as employees struggle to maintain their psychological well-being and engagement in the face of relentless technological changes and expectations. Based on the above theories, this study proposes the following hypothesis.

**Hypothesis** **2.** *AI-induced stress has a significant negative effect on psychological capital*.

Past studies have shown that psychological capital is positively related to hotel employees’ happiness, work engagement, and sense of professional identity, while it is negatively related to burnout and turnover intention [29,30,31]. In addition, hope, optimism, and resilience, combined with psychological capital, have stronger positive correlations with their job performance [29]. Based on the above findings, the following hypotheses are proposed in this study. The COR theory, proposed by Hobfoll, suggests that individuals protect and accumulate resources when faced with stress. Psychological capital—comprising self-efficacy, optimism, resilience, and hope—plays a significant role in enhancing employees’ ability to cope with workplace challenges, thereby positively impacting work engagement. Specifically, heightened self-efficacy encourages employees to actively participate in their tasks and persevere through difficulties, while optimism fosters a problem-solving attitude that helps them focus on the positives of their work. Additionally, resilience allows employees to recover quickly from setbacks, maintaining their focus and reducing emotional disturbances that could hinder engagement. Hope, as a crucial element, motivates employees to set and pursue goals, leading to increased proactivity and involvement. Overall, by strengthening these components of psychological capital, organizations can effectively promote higher levels of employee engagement and enhance overall performance.

**Hypothesis** **3.** *Psychological capital has a significant positive effect on work engagement.*

COR theory suggests that individuals will attempt to conserve and acquire various resources in the face of stress, including physical, social, emotional, and cognitive resources [11]. Psychological capital is a type of internal resource that is believed to help an individual cope with stress and achieve success, including self-confidence, optimism, self-discipline, and resilience [12]. At the same time, psychological capital can enhance the competitiveness of an organization by increasing employees’ job satisfaction, work engagement, and job performance [29]. It has been suggested that psychological capital can mediate the relationship between job stress and work engagement [32]. For example, scholars have found that psychological capital can help employees cope with work stress and improve their performance and innovative behaviors [33]. Specifically, self-confidence, optimism, and resilience in psychological capital can help hotel employees cope with work stress more effectively and maintain positive work attitudes and behaviors. Meanwhile, scholars believe that when individuals have a high level of psychological capital, they tend to remain positive and optimistic in the face of difficulties and are willing to take the initiative to overcome the current difficulties. Based on the above inferences, this study proposes the following hypothesis.

**Hypothesis** **4.** *Psychological capital mediates the effect of AI-induced stress on work engagement.*

### 2.3. Moderating Role of Perceived Organizational Support Between AI-Induced Stress and Psychological Capital

Social support theory suggests that the social support perceived by individuals can have a significant impact on their ability to cope with stress and adapt to their environment. This theory encompasses various forms of support, including emotional, informational, and tangible assistance, which collectively contribute to one’s resilience in the face of adversity [16]. In particular, perceived organizational support—defined as the extent to which employees believe their organization values their contributions and cares about their well-being—can play a crucial role in this dynamic. By fostering a positive workplace culture, organizations can offer emotional comfort through empathetic leadership and supportive peer networks, which are essential for enhancing employees’ mental health. Additionally, providing informational guidance through training programs and resources enables individuals to navigate the complexities and uncertainties introduced by AI technologies, thereby reducing anxiety and enhancing clarity. Moreover, access to various resource assistance, such as mental health services or stress management workshops, equips individuals with practical tools and strategies to better cope with AI-induced stressors. Consequently, a strong sense of perceived organizational support not only bolsters employees’ psychological well-being but also contributes to a more adaptive and resilient workforce, ultimately leading to improved organizational performance and employee satisfaction [34].

Perceived organizational support may modulate the effects of AI-induced stress on psychological capital through multiple mechanisms. First, perceived organizational support can alleviate the negative effects of AI-induced stress on individuals’ psychological capital and increase individuals’ self-efficacy and positive emotions [35]. The higher the perceived organizational support, the greater the mitigating effect of perceived organizational support on the negative effects of AI-induced stress on psychological capital, the greater the increase in self-efficacy and positive emotions of hotel employees, and the greater the psychological capital of hotel employees [36]. Secondly, perceived organizational support can enhance individuals’ ability to cope with stress and promote the construction and maintenance of their psychological capital. The higher the perceived organizational support, the better the construction and preservation of self-efficacy, hope, optimism, and resilience of individual hotel employees’ psychological capital, and the less likely that AI-induced stress will have a negative impact on psychological capital [17]. Finally, perceived organizational support may help individuals cope with stress more effectively by providing resources and help, thus maintaining the stability of psychological capital. The higher the perceived organizational support, the more resources and help are provided to hotel employees. The higher the perceived organizational support, the more resources and help it provides to hotel employees. When hotel employees face AI-induced stress, they are more able to cope with it positively, thus maintaining the stability of psychological capital [37].

When individuals experience high levels of AI-induced stress, organizational support can alleviate the negative effects of stress and promote the development of individuals’ psychological capital. Specifically, organizational support can help individuals cope with stress, enhance their self-confidence, optimism, and resilience, and, thus, increase their psychological capital [38]. Scholars have found that organizational support and psychological capital can effectively reduce the negative impact of work stress, enhance the level of happiness, and play an important role in meeting the material and spiritual needs of employees [39]. In addition, when individuals feel the support provided by the organization, they are more likely to feel social support and more likely to cope with AI-induced stress, thus enhancing their psychological capital [40]. In summary, the stronger the perceived organizational support of employees, the stronger the effect of AI-induced stress in moderating the negative effect of AI-induced stress on psychological capital. Based on the above theories, the following hypothesis was proposed in this study.

**Hypothesis** **5.** *Perceived organizational support moderates the relationship between AI-induced stress and psychological capital. Specifically, the stronger the perceived organizational support, the stronger the positive effect of AI-induced stress on psychological capital.*

**Figure 1:**
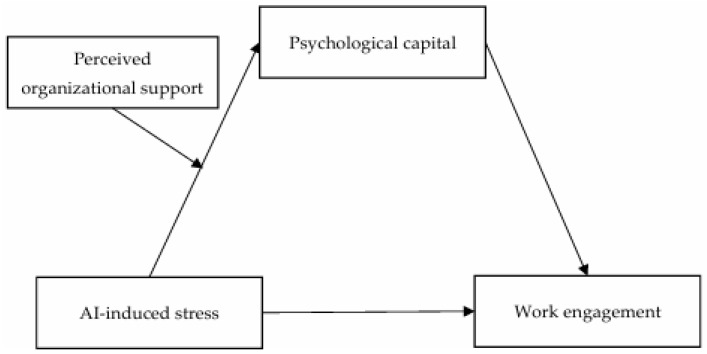
Research model.

In summary, the research model diagram of this paper is shown in Figure 1.

## 3. Method

### 3.1. Data Collection

In this paper, we study the employees of five-star hotels in China. First, five-star hotels commonly apply various AI technologies (e.g., intelligent customer service, data analytics, intelligent recommendation systems, etc.), which directly affect employees’ daily workflow and task requirements, causing them to face new skill needs and adjustments to their work styles. This change brings specific pressure to use AI, different from other hotel employee groups that have not widely adopted AI. Second, five-star hotels have extremely high demands on service quality and customer experience, and this demanding work environment makes employees more susceptible to additional pressures due to AI use. For example, AI-driven performance tracking systems and real-time customer feedback mechanisms place higher demands on employee skills and adaptability. In this environment, the use of AI not only raises customer expectations but also makes employees feel a greater sense of being supervised and under time pressure, resulting in unique work pressures associated with AI use. Finally, the work environment in five-star hotels does place higher demands on employees‘ competencies and behaviors, but this is the significance of the study’s selection of this group: the impact of AI on employees’ stress is particularly pronounced in a higher standard of service environment, and the study was able to capture more clearly the work-related stress due to AI use.

These hotels fulfill the following criteria: first, the employees work with AI in their work. For example, many hotels use AI-powered virtual concierges (such as chatbots or voice assistants) to provide 24/7 service to guests. For example, when guests want to learn about local attractions, book a restaurant, or obtain transport information, they can communicate with the virtual concierge via their mobile phone or in-room smart device. Hotel staff, in turn, can manage and optimize the AI’s service offerings to ensure guests receive accurate responses and reduce their own duplication of effort. Second, these hotels are five-star hotels. Third, these hotels have introduced AI for more than half a year. After the questionnaire design was completed, the project team members imported the questionnaire into Questionnaire Star and generated the links. We cooperated with the heads of the relevant hotel companies, and through communication and negotiation with the heads of the relevant hotels, we filled in the e-questionnaire during the staff’s breaks.

We used our alumni resources to contact 12 hotels in China. To minimize the effect of common methodological bias on the data, we collected data at two time points. In April 2024, we first collected AI-induced stress, perceived organizational support, and demographic variables. A total of 330 questionnaires were collected, and 301 valid questionnaires were returned. In May 2024, we also collected psychological capital and work engagement. In total, 301 questionnaires were distributed, and 238 were returned. We excluded the questionnaires that were missed and obviously wrong and finally obtained 209 valid questionnaires. In order to ensure that the data can be matched accurately, each time they fill out the questionnaire, the subjects have to fill in the last six digits of the cell phone number. Specifically, our criteria for excluding poor-quality questionnaires were as follows: first, incomplete questionnaires. If more than a certain percentage of the questions in the questionnaire were not answered (e.g., more than 20 percent of the questions were left blank), we considered the questionnaire incomplete and excluded it. Second, extreme or unreasonable answer times. Questionnaires that took too short a time to complete (e.g., less than the set minimum response time) indicate that they may have been answered randomly. We judge this by the response times recorded by the questionnaire system. For example, if the average response time was 15 min, a questionnaire with a response time below 5 min could be regarded as not being answered seriously. Third, repeated answers to the same option. Choosing the same answer for all questions (e.g., “strongly agree” or “strongly disagree” for all of them) may indicate that the participant has not thought hard enough. This “consistency bias” is often seen as a sign of non-seriousness. Fourth, responses were not logically consistent. Participants give inconsistent answers to logically related questions. For example, they give contradictory answers to positive and negative descriptions of the same concept. In addition, “obvious errors” in the completion of the questionnaire were as follows. Firstly, answers beyond the reasonable range. For example, when filling in basic information such as age, length of service, education, etc., the answers were clearly beyond the reasonable range (e.g., employees under 15 years old, more than 100 years of service, etc.). Second, invalid answers to open-ended questions. Subjects fill in meaningless letters, symbols, or irrelevant content in open-ended questions (e.g., “abcd”, “meaningless word”, etc.). Third, intentionally giving untrue information. Subjects answering “untrue” or “test only” for questions that directly ask about the truth of the information indicates that the questionnaire is not trustworthy and should be excluded. The basic characteristics of the sample are shown in Table 1.

### 3.2. Measurement

The present study was conducted on the well-established scales borrowed from domestic and foreign scholars. The measurement scales were based on a five-point Likert scale, with values ranging from 1 to 5 for “strongly disagree” to “strongly agree”, respectively. Please see Appendix A for question items for all variables.

For the measurement of AI-induced stress, the present study drew on a scale developed by Gaudioso to measure AI-induced stress [41]. The sample questions are “I spend less time with my family due to AI” and “I have a higher workload because of increased AI complexity”. The scale was self-administered by employees.

For the measurement of work engagement, this study drew on the 18-item JES scale developed by Rich et al. in 2010 [42]. The sample questions are “I work with intensity on my job” and “I exert my full effort to my job”. The scale was self-administered by employees.

For the measurement of psychological capital, the present study drew on the scale developed by Gorgens-Ekermans and Herbert to measure psychological capital [43]. The sample questions are “I believe that I can analyze long-term problems and find solutions” and “When I have a meeting with management, I am confident in stating things that are within my scope of work”. The scale is self-administered by the employee.

For the measurement of perceived organizational support, this study drew on the eight-item scale developed by Shen and Benson to measure perceived organizational support [44]. The sample questions are “My organization cares about my well-being” and “My organization cares about my opinions”. The scale was self-rated by employees.

Control variables: drawing on the findings of previous scholars, this paper chooses gender, age, education, marital status, tenure, and workplace as control variables [10].

## 4. Results

### 4.1. Reliability Analysis

In this paper, the four variables of AI-induced stress, work engagement, psychological capital, and organizational support were tested for reliability using SPSS 27.0 software, and the test results are shown in Table 2. The Cronbach’s coefficient for AI-induced stress is 0.932, the Cronbach’s coefficient of engagement is 0.864, the Cronbach’s coefficient of perceived organizational support is 0.847, and the Cronbach’s coefficient of psychosocial capital is 0.904. The result is greater than 0.8, which shows that the indexes of each scale in the thesis are reliable to a certain extent. The results are all greater than 0.8, which shows that the indicators of each scale in this thesis are reliable to a certain extent and have a high degree of consistency in measuring the target concepts or attributes, i.e., they have good reliability.

### 4.2. Validity Analysis

Validity testing is the process of evaluating the validity of a measurement instrument. It seeks to determine whether the instrument accurately measures the concept or characteristic it purports to measure. In order to test the discriminant validity among variables, this study conducted a validation factor analysis using Mplus 7.4 analysis software. Specifically, this study tested the discriminant validity of the variables by comparing the goodness-of-fit indices of the baseline model (four-factor) with those of the competing models (three-factor, two-factor, and one-factor). The test results are shown in Table 3.

The four-factor model is AI-induced stress, work engagement, psychological capital, and perceived organizational support; the three-factor model is AI-induced stress, work engagement, and psychological capital + perceived organizational support; the two-factor model is AI-induced stress + work engagement, psychological capital + perceived organizational support, and psychological capital + perceived organizational support; the one-factor model is AI-induced stress + work engagement + psychological capital + perceived organizational support. As shown in Table 3, the four-factor model (χ2/df = 2.418, RMSEA = 0.083, TLI = 0.897, CFI = 0.91, SRMR = 0.064) has all matching indices at acceptable levels, and its fitting effect is better than the other competing models, so the four variables used in this study have good discriminant validity.

### 4.3. Correlation Analysis

Pearson’s correlation coefficient was used to indicate the strength of the correlation. Pearson’s method was chosen by averaging the items of the variable as the value of the variable and bringing the four variables into the correlation analysis.

As can be seen from Table 4, there is a certain degree of correlation between AI-induced stress, work engagement, psychological capital, and perceived organizational support. The correlation coefficient between AI-induced stress and psychological capital is −0.290 and shows significance at the 0.01 level, thus indicating that there is a significant negative correlation between AI-induced stress and psychological capital, which suggests that when individuals face excessive AI-induced stress, their psychological capital will be reduced. This suggests that when individuals face excessive AI-induced stress, their level of psychological capital may decrease, affecting their coping ability and work performance. The correlation coefficient between AI-induced stress and work engagement is −0.390 and shows significance at the 0.01 level, which indicates that there is a significant negative correlation between AI-induced stress and work engagement, which indicates that excessive AI-induced stress might weaken the individual’s motivation, attention, emotional engagement and creativity, and, thus, reduce the degree of commitment to the work and the level of performance.

The correlation coefficient between AI-induced stress and psychological capital is −0.29, which is statistically significant but explains a low variance. We believe that the result still has important theoretical and practical significance in the following ways: first, psychological capital, as a positive psychological resource, may partially mitigate employees’ AI-induced stress, but it cannot completely eliminate it. This negative correlation of −0.29 suggests that although psychological capital can help employees adapt to the changes and challenges due to AI to a certain extent, AI-induced stress is still significantly influenced by other factors, such as the work environment and individual familiarity with technology. Second, although the explained variance is less than 9%, the correlation coefficient reflects the role of psychological capital as a “buffer” against AI-induced stress. Such a relationship provides insights for managers that, in the context of widespread AI adoption, it is effective in enhancing employees’ psychological capital (e.g., increasing self-confidence and stress tolerance) to reduce their AI-induced stress. In addition, this result provides a direction for subsequent studies to further explore other possible influencing factors. Finally, we also considered the correlation coefficient between psychological capital and AI-induced stress, although not very high, and similar negative correlations are usually considered to be of practical value in employee adaptation and mental health research. Therefore, we believe that this result can provide a valuable reference for interventions in the hotel industry during AI adoption.

### 4.4. Hypothesis Testing

#### 4.4.1. Regression Analysis of AI-Induced Stress on Work Engagement

According to the results in the Table 5, the R2-explained variation decreased from 24.1% to 21.5%, indicating that AI-induced stress explained 2.6% of the variation in work engagement. Moreover, the coefficient of AI-induced stress showed −0.324 at a 0.05 significant level, and the significance was confirmed. The results of the study show that AI-induced stress has a significant negative effect on work engagement. Hypothesis 1 was supported.

#### 4.4.2. A Test of the Mediating Role of Psychological Capital in AI-Induced Stress and Work Engagement

(1) Test Hypothesis 2: AI-induced stress has a significant negative effect on psychological capital. AI-induced stress was analyzed by linear regression with AI-induced stress as the independent variable, psychological capital as the dependent variable, and demographic variables as the control variables.

It can be seen in Table 6 that when AI-induced stress was introduced into the regression equation, the coefficient of AI-induced stress was −0.286 at the level of 0.05, which had a significant effect, and the R^2^-explained variance was reduced from 13% to 10%, indicating that AI-induced stress explained 3% of the variance of the work engagement. The results show that AI-induced stress has a significant negative effect on psychological capital. Hypothesis 2 was supported.

(2) Test Hypothesis 3: Psychological capital has a significant positive effect on work engagement. A linear regression analysis was conducted with psychological capital as the independent variable, work engagement as the dependent variable, and demographic variables as the control variables.

From the results of the Table 7, it can be seen that when psychological capital is introduced into the regression equation, the coefficient of psychological capital is 0.477 at the level of 0.05, which has a significant effect, and the R2 explains the variation from 41.7% to 39.6%, which indicates psychological capital in the regression equation. The results show that AI-induced stress has a significant positive effect on psychological capital. Hypothesis 3 was supported.

(3) Testing Hypothesis 4: Psychological capital mediates the effect of AI-induced stress on work engagement. This study, based on the COR theory, concluded that AI-induced stress may lead to emotional exhaustion of individuals, thus affecting their work engagement, i.e., when individuals are subjected to AI-induced stress for a long period of time, they may experience emotional exhaustion, fatigue, and feelings of helplessness. This kind of emotional exhaustion will make the individual feel low energy and low mood at work and even lose enthusiasm and motivation for work. This emotional exhaustion affects an individual’s cognitive and affective resources and reduces his or her commitment and attention to work tasks. The patterns of mediating effects can be further categorized into regression models: Type 1 regression models are regression analyses of AI-induced stress and work engagement; Type 2 regression models are regression modeling of AI-induced stress with demographic variables; Type 3 regression models are regression modeling of AI-induced stress and demographic variables together with work engagement. The results of the data analysis are shown in Table 8.

From the results of the Table 9, it can be seen that AI-induced stress has a negative effect on work engagement and a positive effect indirectly through psychological capital, i.e., the mediating effect of psychological capital partially exists. Therefore, Hypothesis 4 was supported.

#### 4.4.3. The Moderating Role of Perceived Organizational Support

The results of the data analysis are shown in Table 10. Firstly, the variables of AI-induced stress, work engagement, psychological capital, and perceived organizational support were centered. The moderated effect study is divided into three models, which are described as follows: Model 1 analyzes the effect of centered AI-induced stress on work engagement; Model 2 adds centered perceived organizational support on the basis of Model 1; Model 3 adds centered AI-induced stress and centered perceived organizational support interaction term; if the change of F-value is significant when changing from Model 2 to Model 3, it implies that there is a moderating effect; if the interaction term shows significance in Model 3, it implies that there is a moderating effect.

The results of the analysis showed that the interaction item with a *p*-value of 0.000 and less than 0.05 affected work engagement, indicating that between AI-induced stress and psychological capital, organizational support moderated the effect significantly. Since the moderating interaction item with b = 0.430 belongs to the positively moderating negative effect, the negative effect of AI-induced stress on psychological capital is weaker when perceived organizational support is higher. Hypothesis 5 was supported.

## 5. Discussion

From the results of the study, it can be concluded that AI-induced stress has a negative effect on extra engagement. The conclusion can be elaborated from several perspectives: AI-induced stress may lead to mental health problems for hotel employees, and long-term AI-induced stress may lead to hotel employees’ lack of positive work attitudes and motivation, which may affect work engagement. Long-term AI-induced stress may also have a negative impact on the physical health of hotel employees, and physical health problems may cause hotel employees to feel tired and weak, reducing work efficiency and engagement; high-intensity AI-induced stress may lead to tense interpersonal relationships among hotel employees, in which case employees may feel emotionally affected and unable to fully concentrate on their work, thus affecting work engagement. This result suggests that it is reasonable to conclude that AI-induced stress negatively affects work engagement. Effective management and reduction of AI-induced stress to improve the working environment and mental health of hotel employees is important for improving work engagement.

Artificial intelligence (AI) is transforming the hospitality industry by streamlining operational processes and improving service quality. However, the introduction of AI technology has also led to employees facing AI-induced stress. This stress usually manifests itself in a number of ways. Employees may feel anxious about rapid technological changes and worry that they lack the skills needed to adapt to new systems. This uncertainty can lead to them feeling less self-worth and less confidence in their job performance. For example, a receptionist using an AI booking system may worry about making mistakes during peak check-ins, which could affect customer satisfaction and lead to greater stress. In addition, AI-induced stress often stems from coping with high expectations set by the AI system. Employees may feel that their work is constantly monitored and evaluated by algorithms, which, in turn, creates a sense of being supervised. This feeling may lead to a disconnect between employees and customers, as employees may be more focused on the efficiency of AI and neglect the personal attention that customers expect in hospitality services. Common employee complaints include frustration with a lack of control over workflow and fear of being replaced by technology. For example, employees may fear job insecurity as their role will diminish as AI takes on tasks traditionally performed by humans. In addition, relying on AI for decision-making may lead to employees feeling a loss of autonomy, and they may feel that their professional judgment is undermined by algorithmic advice. This may produce cognitive dissonance, with employees feeling more stress as they try to reconcile their expertise with the AI recommendations. In discussions among colleagues, employees often share their anxiety about how AI is changing work roles and responsibilities, emphasizing the need for training and support in this technological environment. In conclusion, AI-induced stress is a multifaceted phenomenon with implications for employee well-being and performance. Understanding its manifestations and the contexts in which it arises is essential for developing strategies to mitigate this stress and creating supportive work environments.

The results of the study show that psychological capital plays a mediating role in the effect of AI-induced stress on work engagement, which suggests that psychological capital has an important significance in the effect of AI-induced stress on work engagement. Combined with the COR theory and the concept of psychological capital, it can be known that the effects of AI-induced stress on work engagement can be explained by the individual’s emotional resources and psychological capital. Emotions, as a kind of resource, may be violated and depleted in the face of AI-induced stress, resulting in the reduction of individuals’ emotional resources. When an individual’s emotional resource is violated, in order to protect this resource, the individual may reduce his/her physical and psychological inputs, including communication at home and positive inputs at work. However, psychological capital, as a positive psychological resource, can alleviate the depletion and protection of emotional resources to a certain extent, thus facilitating individuals to re-engage in their work. Therefore, if the organization can recognize the importance of hotel employees’ emotions and psychological capital in management and respond to hotel employees’ adverse emotions in a timely manner, provide support and resources to help hotel employees recover their emotional resources, and enhance their psychological capital, it can promote hotel employees to re-engage in their work. This suggests that psychological capital plays a mediating role in the effects of AI-induced stress on work engagement and influences the level of individual work engagement by enhancing the individual’s psychological resources and regulating the emotional state.

The test results in this paper indicate that perceived organizational support positively moderates the relationship between AI-induced stress and psychological capital. AI-induced stress may have a negative impact on an individual’s psychological capital. Prolonged AI-induced stress may cause individuals to feel tired, helpless, and unmotivated, which may diminish the positive factors of their psychological capital. Perceived organizational support refers to the degree of support and perceived support resources of an organization. When individuals perceive the support provided by the organization, it will enhance their sense of identity and belonging to the organization and, thus, increase their psychological capital. Meanwhile, psychological capital can help individuals to cope with challenges, maintain a positive attitude, and improve their adaptive ability. Therefore, perceived organizational support can play a positive moderating role between AI-induced stress and psychological capital and enhance individuals’ psychological capital by providing support resources and care. When an individual faces stress at work, if he/she feels the support of the organization, it will enhance his/her confidence and ability to cope with the stress, which, in turn, will enhance the positive factors of psychological capital, such as self-efficacy, hope, and optimism, and help the individual cope with the AI-induced stress better and maintain a positive attitude and commitment to his/her work.

### 5.1. Theoretical Contributions

First, this study enriches the work stress literature and the research on antecedent variables of work engagement. Based on the COR theory and social support theory, this study considers AI-induced stress as a negative factor that can easily lead to the depletion of their own resources at work, and finally, it will have an impact on the hotel employees’ work engagement from the perspective of resource depletion, explaining the intrinsic effect of AI-induced stress on work engagement in the hotel industry. Finally, AI-induced stress will have an impact on hotel employees’ engagement, which explains the intrinsic mechanism of AI-induced stress on work engagement in the hotel industry. This study investigates the impact of AI-induced stress on work engagement, which enriches the research on antecedent variables of work engagement. In addition, recent studies on the consequences of work stress have mainly focused on the performance of hotel employees [45,46]. This study clarifies the mechanism of AI-induced stress and work engagement and enriches the antecedent variables and research scenarios of work engagement.

Second, the present study enriches the research on psychological capital. Psychological capital plays an important role in employee work and is often seen as an important variable in explaining employee behavior. Previous research on the mediating mechanism between AI-induced stress and its outcome variables has been insufficient, and the relevant variables have mainly focused on emotional exhaustion [47]. The present study strengthens the understanding of the relationship between AI-induced stress and work engagement. Psychological capital is not only an individual’s resource but also an organizational resource, which is crucial for organizational development and performance [26]. Therefore, it is meaningful to explore the mediating role of psychological capital in the mechanism of AI-induced stress on work engagement, which enriches the research on psychological capital as a mediating variable.

Third, the present study used a sense of organizational support as a moderating variable, which strengthens the understanding of the boundary conditions for the effects of AI-induced stress. Perceived organizational support has often been used as a mediating variable in previous studies to examine its effects on employee behavior [48,49]. Current research has explored some of the boundary conditions, such as colleagues’ uncivil behavior and transformational leadership, under which AI-induced stress enhances or attenuates the effects on hotel employees [50]. In the present study, perceived organizational support was considered an important moderating variable, and it was added to the models of AI-induced stress, psychological capital, and work engagement to investigate the effects of different levels of perceived organizational support on AI-induced stress, work engagement, and organizational support. Induced stress, work engagement, and psychological capital deepen the research on perceived organizational support.

### 5.2. Practical Implications

First, organizations should pay attention to the negative impact of AI-induced stress on work engagement, which contributes to the core competitiveness and sustainability of enterprises. Research has shown that AI-induced stress reduces the motivation of individuals to work actively and prevents them from continuously devoting energy and time to their work. Excessive or prolonged AI-induced stress may lead to fatigue, anxiety, and boredom, reducing the level of commitment of hotel employees to their work. Prolonged AI-induced stress may lead to burnout and affect the motivation of work engagement. This study found that AI-induced stress can significantly and negatively affect work engagement, and the conclusion helps managers recognize the harm of excessive AI-induced stress. When hotel employees feel excessive AI-induced stress, enterprises should improve their psychological state by formulating reasonable incentive mechanisms, providing a good working environment, and strengthening the training and development of hotel employees so as to promote the sustainable development of the enterprise.

Second, this study helps managers to emphasize and pay close attention to the changes in the psychological attitudes of hotel employees. Specifically, they can introduce targeted training programs focusing on resilience, stress management, and emotional intelligence to equip employees with essential coping skills. Regular psychological assessments through surveys or one-on-one meetings can help identify stressors and gauge psychological capital levels, allowing for timely interventions. Establishing Employee Assistance Programs (EAPs) can provide counseling and support for those experiencing mental health challenges. Additionally, fostering a positive work culture that encourages open communication and recognition, along with offering flexible work arrangements, can significantly reduce stress and increase employee satisfaction. Implementing wellness initiatives, such as mindfulness training and relaxation techniques, further promotes psychological health. Training leaders to recognize and address changes in employee attitudes will empower them to support their teams effectively. Creating a feedback mechanism for employees to voice concerns related to AI-induced stress can inform management practices while setting clear expectations about roles and responsibilities can alleviate uncertainty. Lastly, organizing team-building activities to strengthen workplace relationships will enhance camaraderie and create a more supportive environment. By adopting these measures, hotel managers can effectively support their employees in managing AI-related stress, ultimately improving work engagement and overall organizational performance.

Third, this study helps managers adopt human resource management strategies to build a supportive culture and climate based on the finding that perceived organizational support plays a positive moderating role between AI-induced stress and psychological capital. Companies can enhance perceived organizational support by fostering a supportive, understanding, and caring culture for employees, which may include encouraging interactions between leaders and coworkers, providing opportunities for employees to participate in decision-making, and establishing open communication channels. Additionally, implementing regular training sessions on stress management and resilience can equip employees with the skills to cope with AI-induced challenges. Providing mental health resources, such as access to counseling services or wellness programs, can further support employees in maintaining their psychological well-being. From the perspective of social support, perceived organizational support can help hotel employees alleviate the negative effects of AI-induced stress on psychological capital, ultimately leading to a more engaged and productive workforce.

### 5.3. Research Limitations and Future Directions

In this paper, although the results of hypothesis testing and empirical testing are basically consistent, there are some research limitations. First of all, the number of studies in this paper is limited, with a valid research sample of only 209 points, indicating that the data may not be very sufficient to draw broad conclusions. A larger and more diverse sample size is essential for enhancing the reliability and generalizability of the findings. Future studies can employ more effective methods in the distribution and collection of questionnaires to ensure that sufficient data are gathered, such as utilizing online survey platforms that can reach a wider audience or conducting longitudinal studies to track changes over time. Secondly, the measures of AI-induced stress, work engagement, perceived organizational support, and psychological capital typically rely on individuals’ self-reports, which may introduce subjectivity and be susceptible to memory bias. To mitigate these concerns, future research could integrate objective data and assessment methods, such as physiological measures of stress or observational assessments of work engagement, to improve both the objectivity and accuracy of these measurements.

Meanwhile, future research can investigate cultural nuances by comparing the differences in AI-induced stress, work engagement, and perceived organizational support across various cultural contexts, as well as examining the role of psychological capital within these different frameworks. This cross-cultural perspective could significantly enhance the breadth and depth of the study. Additionally, intervention studies focusing on AI-induced stress and perceived organizational support could be conducted to explore practical approaches for improving the level of work engagement among hotel employees, such as modifying the work environment or providing targeted support programs. Finally, beyond the mediating role of psychological capital, future research should consider exploring other potential moderating variables and mediating mechanisms, such as workplace climate, leadership styles, or individual personality traits. This comprehensive approach could offer deeper insights into the complex processes through which AI-induced stress impacts work engagement, ultimately contributing to more effective management strategies in the hospitality industry.

## 6. Conclusions

This study provides valuable insights into the impact of AI-induced stress on hotel employees’ work engagement, emphasizing the mediating role of psychological capital and the moderating role of perceived organizational support. By applying the Conservation of Resources (COR) theory and social support theory, we reveal that AI-induced stress can significantly decrease work engagement among employees in five-star hotels in China. However, this negative effect is partly buffered by psychological capital, which serves as a mediating factor. Furthermore, perceived organizational support plays a crucial role in moderating the relationship between work stress and psychological capital. Specifically, when employees perceive higher organizational support, the detrimental effects of work stress on their psychological capital are reduced, thus indirectly supporting their work engagement. These findings contribute to the growing body of research on AI’s implications in the hospitality industry, highlighting both the challenges and strategic responses that managers can adopt. For practitioners, this study underscores the importance of fostering psychological capital and enhancing perceived organizational support to help employees adapt to AI-driven changes. By doing so, hotel managers can mitigate the negative effects of AI-induced stress, ultimately promoting a more resilient and engaged workforce amidst the ongoing integration of AI in hotel operations.

## Figures and Tables

**Table 1:** Demographic characteristics.

Variable	Categorization	Percentage	Variable	Categorization	Percentage
gender	male	58.37%	tenure	3 years and under	16.27%
	women	41.63%		3–5 years	52.63%
age	under 20	0.00%		5–10 years	21.05%
	21–30 years	38.28%		more than 10 years	10.05%
	31–40 years	44.02%	workplace	technical staff	34.45%
	41–50 years	14.83%		administrator	29.67%
	over 50 years old	2.87%		grassroots management	16.27%
education	high school and below	11.96%		middle management	13.40%
	specialized training school	24.88%		senior management	6.22%
	undergraduate (adjective)	50.72%	marital status	unmarried	38.28%
	bachelor’s degree	10.05%		married	51.67%
	doctoral	2.39%		(sth. or sb) else	10.05%

**Table 2:** Results of scale reliability test.

Variable	Cronbach’s Alpha Coefficient
AI-induced stress	0.932
Work engagement	0.864
Perceived organizational support	0.847
Psychological capital	0.904

**Table 3:** Results of validation factor analysis.

Model	χ2	df	χ2/df	RMSEA	CFI	TLI	SRMR
Four-factor	442.491	183	2.418	0.083	0.91	0.897	0.064
Three-factor	762.802	186	4.101	0.122	0.8	0.774	0.105
Two-factor	1274.632	188	6.78	0.167	0.623	0.579	0.179
One-factor	1780.674	189	9.422	0.201	0.447	0.386	0.194

**Table 4:** Results of correlation analysis.

Variable	Mean	SD	1	2	3	4
1. AI-induced stress	3.394	1.022	1			
2. Psychological capital	3.346	1.112	−0.290 **	1		
3. Perceived organizational support	3.122	1.13	−0.065	0.304 **	1	
4. Work engagement	3.906	0.948	−0.390 **	0.594 **	0.396 **	1

Note: ** *p* < 0.01.

**Table 5:** Regression analysis of AI-induced stress on work engagement.

Variable	Non-Standardized Coefficient		Standardized Coefficient	t	*p*
	B	SE	Beta		
Gender	0.403	0.122	0.21	3.312	0.001 **
Age	0.048	0.155	0.039	0.307	0.759
Education	0.128	0.067	0.122	1.93	0.055
Marital status	0.043	0.138	0.029	0.315	0.753
Tenure	−0.223	0.115	−0.199	−1.929	0.055
Workplace	−0.063	0.049	−0.083	−1.289	0.199
AI-induced stress	−0.324	0.058	−0.35	−5.609	0.000 **
R^2^	0.241				
Adjustment of R^2^	0.215				
F	F (7,201) = 9.131, *p* = 0.000				
D-W value	1.901				

Note: ** *p* < 0.01.

**Table 6:** Regression analysis of AI-induced stress on psychological capital.

Variable	Non-Standardized Coefficient		Standardized Coefficient	t	*p*
	B	SE	Beta		
Gender	0.114	0.153	0.051	0.747	0.456
Age	0.252	0.194	0.178	1.296	0.196
Education	0.181	0.084	0.146	2.158	0.032 *
Marital status	−0.015	0.173	−0.008	−0.085	0.932
Tenure	−0.272	0.145	−0.207	−1.878	0.062
Workplace	0.021	0.062	0.024	0.348	0.728
AI-induced stress	−0.286	0.073	−0.263	−3.938	0.000 **
R^2^	0.13				
Adjustment of R^2^	0.1				
F	F (7,201) = 4.307, *p* = 0.000				
D-W value	1.961				

Note: * *p* < 0.05, ** *p* < 0.01.

**Table 7:** Regression analysis of psychological capital on work engagement.

Variable	Non-Standardized Coefficient		Standardized Coefficient	t	*p*
	B	SE	Beta		
Gender	0.388	0.106	0.202	3.644	0.000 **
Age	−0.027	0.136	−0.023	−0.201	0.841
Education	0.056	0.059	0.053	0.95	0.343
Marital status	0.008	0.12	0.005	0.068	0.946
Tenure	−0.123	0.102	−0.11	−1.209	0.228
Workplace	−0.077	0.043	−0.101	−1.797	0.074
Psychological capital	0.477	0.047	0.56	10.067	0.000 **
R^2^	0.417				
Adjustment of R^2^	0.396				
F	F (7,201) = 20.509, *p* = 0.000				
D-W value	2.002				

Note: ** *p* < 0.01.

**Table 8:** Test process of psychological capital’s mediating role in AI-induced stress and work engagement.

Variable	Work Engagement					
	B	t	B	t	B	t
Gender	0.403 **	3.312	0.114	0.747	0.354 **	3.436
Age	0.048	0.307	0.252	1.296	−0.06	−0.457
Education	0.128	1.93	0.181 *	2.158	0.051	0.903
Marital status	0.043	0.315	−0.015	−0.085	0.05	0.427
Tenure	−0.223	−1.929	−0.272	−1.878	−0.106	−1.08
Workplace	−0.063	−1.289	0.021	0.348	−0.072	−1.743
AI-induced stress	−0.324 **	−5.609	−0.286 **	−3.938	−0.202 **	−3.982
Psychological capital					0.427 **	8.987
R^2^	0.241		0.13		0.46	
Adjustment of R^2^	0.215		0.1		0.438	
F-value	f(7,201) = 9.131		f(7,201) = 4.307		F (8,200) = 21.254	

Note: * *p* < 0.05, ** *p* < 0.01.

**Table 9:** Results of the test for the mediating role of psychological capital in AI-induced stress and work engagement.

Total Effect	a	b	Mediate Effect	95% BootCI	Direct Effect’
−0.324 **	−0.286 **	0.427 **	−0.122	−0.206~−0.057	−0.202 **

Note: ** *p* < 0.01.

**Table 10:** Results of the test of the moderating role of perceived organizational support in AI-induced stress and psychological capital.

Variable	Model 1		Model 2		Model 3	
	B	t	B	t	B	t
Gender	0.114	0.747	−0.011	−0.076	−0.051	−0.395
Age	0.252	1.296	0.277	1.476	0.25	1.566
Education	0.181	2.158	0.131	1.601	0.068	0.974
Marital status	−0.015	−0.085	−0.065	−0.387	0.039	0.275
Tenure	−0.272	−1.878	−0.239	−1.703	−0.221	−1.855
Workplace	0.021	0.348	0.006	0.109	−0.011	−0.214
AI-induced stress	−0.286 **	−3.938	−0.278 **	−3.949	−0.231 **	−3.854
Perceived organizational support			0.258 **	3.894	0.325 **	5.732
R^2^	0.13		0.192		0.42	
Adjustment of R^2^	0.1		0.159		0.393	
F-value	f(7,201) = 4.307		F(8,200) = 5.930		F(9,199) = 15.988,	

Note: ** *p* < 0.01.

## Data Availability

The data are not available due to confidentiality concerns. The data collected for this research consist of survey responses from employees of participating companies. Due to privacy and confidentiality concerns expressed by these participants, we have chosen not to publicly disclose the raw survey data. Respecting the wishes of our subjects is paramount in our research ethics. However, should readers require access to the original data, we welcome inquiries via email directed to the corresponding author. Upon obtaining the necessary consent from the participants, we will be willing to share the data as requested.

## Appendix A

Items of AI-induced stress

1. I spend less time with my family due to AI.
2. I have to be in touch with my work even during my vacation due to AI.
3. I have to sacrifice my vacation and weekend time to keep current on new AI.
4. I feel my personal life is being invaded by AI.
5. I am forced by AI to work much faster.
6. I am forced by AI to do more work than I can handle.
7. I am forced by AI to work with very tight time schedules.
8. I am forced to change my work habits to adapt to AI.
9. I have a higher workload because of increased AI complexity.

Items of work engagement

1. I work with intensity on my job.
2. I exert my full effort to do my job.
3. I devote a lot of energy to my job.
4. I try my hardest to perform well on my job.
5. I strive as hard as I can to complete my job.
6. I exert a lot of energy on my job.
7. I am enthusiastic about my job.
8. I feel energetic about my job.
9. I am interested in my job.
10. I am proud of my job.
11. I feel positive about my job.
12. I am excited about my job.
13. At work, my mind is focused on my job.
14. At work, I pay a lot of attention to my job.
15. At work, I concentrate on my job.
16. At work, I focus a great deal of attention on my job.
17. At work, I am absorbed in my job.
18. At work, I devote a lot of attention to my job.

Items of psychological capital

1. I believe that I can analyze long-term problems and find solutions.
2. When I have a meeting with management, I am confident in stating things that are within my scope of work.
3. I believe that I contribute to the discussion of the company’s strategy.
4. Within the scope of my work, I believe I am able to help set goals/objectives.
5. I believe that I am able to connect with people outside the company (e.g., suppliers, customers) and discuss problems.
6. I believe in being able to present information to a group of colleagues.
7. If I find myself stuck in a rut at work, I can come up with a lot of ways to get out of it.
8. At the moment, I am energetic and energetic enough to complete my work goals.
9. There are many workarounds for any problem.
10. Right now, I consider myself quite successful at work.
11. I can come up with a lot of ways to achieve my current work goals.
12. Currently, I am achieving the work goals I have set for myself.
13. When I have a setback at work, it is hard for me to bounce back and move on. (R)
14. At work, I will solve the problems that come my way.
15. If I have to do it at work, I can say that I can also fight on my own.
16. I usually take the pressure of work in stride.
17. Because I have been through a lot of hardships before, I can now survive the difficult times at work.
18. In my current job, I feel like I can handle a lot of things at the same time.
19. At work, when faced with something uncertain, I usually look forward to the best.
20. If something goes wrong, it will go wrong even if I work wisely. (R)
21. I always see the bright side of things in my work.
22. I am optimistic about what the future holds for my work.
23. In my current job, things have never turned out the way I had hoped. (R)
24. When I work, I always believe that “behind the darkness is the light, there is no need to be pessimistic”.

Items of perceived organizational support

1. My organization cares about my opinions.
2. My organization cares about my well-being.
3. My organization appreciates any extra effort from me.
4. My organization would ignore any complaint from me.
5. Even if I did the best job possible, my organization would fail to notice.
6. My organization cares about my general satisfaction at work.
7. My organization shows very little concern for me.
8. My organization takes pride in my accomplishments at work.
